# Supplementary figures and images for: Lipopolysaccharide transport regulates bacterial sensitivity to a cell wall-degrading intermicrobial toxin
Source: PLoS Pathog. 2023 Jun 26;19(6):e1011454. doi: 10.1371/journal.ppat.1011454 (PMC10328246; doi:10.1371/journal.ppat.1011454)

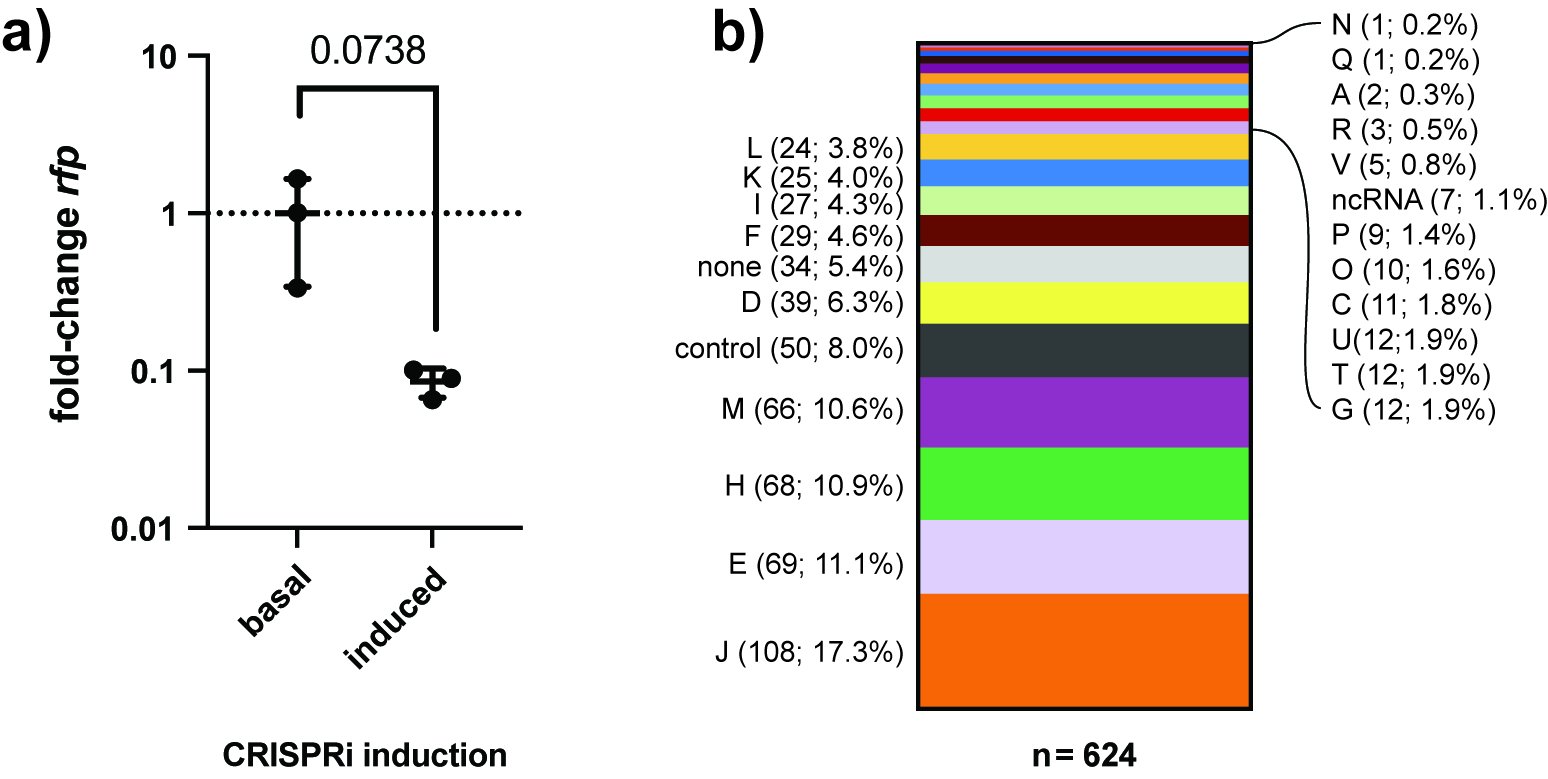

Supplement: S1 Fig — a) CRISPRi induction produces mild transcriptional knockdown of endogenous rfp (11.7-fold decrease) in Eco. qRT-PCR measurement of relative rfp RNA expression in Eco strain SC363 after 6 hours of growth on solid LB media with basal or induced CRISPRi. Data shown: 3 biological replicates with mean ± s.d. Statistical test: unpaired two-tailed t-test. b) CRISPRi targets Eco genes that collectively represent 21 clusters of orthogonal genes (COGs). CRISPRi target genes (n = 596) were binned by their NCBI COG functional assignment. The relative representation of each COG in the strain collection is displayed as a percent of all COGs. Some genes are represented by multiple COGs, resulting in a greater number of COGs (n = 624) than target genes. Non-targeting negative controls (“control”, n = 50) genes without COG assignments (“none”, n = 34), and genes coding for non-coding RNAs (“ncRNA”, n = 7) were also binned. (TIF) [file ppat.1011454.s005.tif]

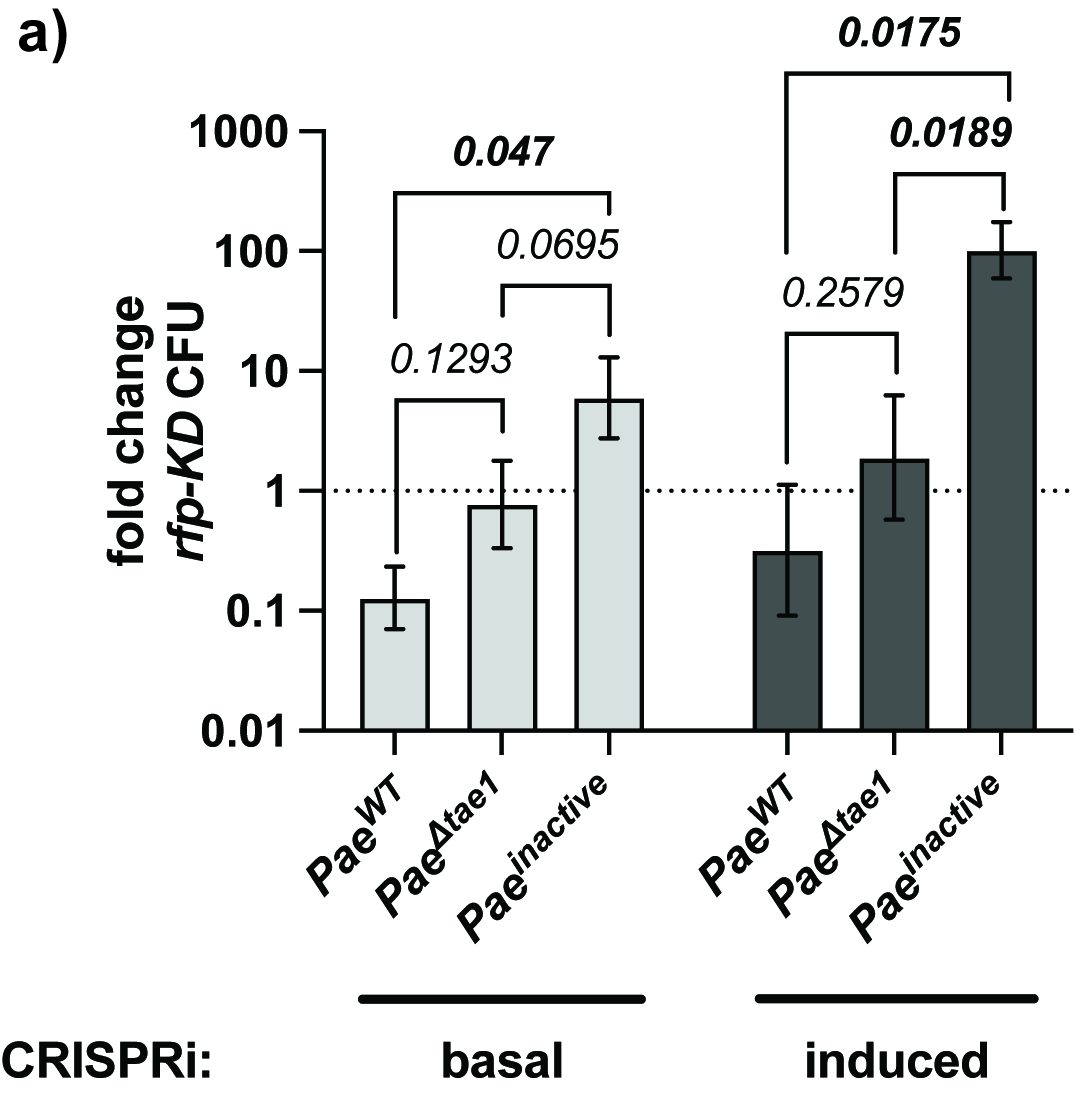

Supplement: S2 Fig — a) CRISPRi induction does not disrupt T6SS- and Tae1-dependent targeting of Eco by Pae. Interbacterial competition between Pae (PaeWT, Pae Δtae1, Paeinactive) and an Eco negative-control KD strain (rfp-KD), with induced or basal CRISPRi. Data shown: mean fold-change (± geometric s.d.) of rfp-KD colony forming units (CFUs) after six hours of competition against Pae. Statistical test: unpaired two-tailed t-test; p-value ≤0.05 displayed in bold font. (TIF) [file ppat.1011454.s006.tif]

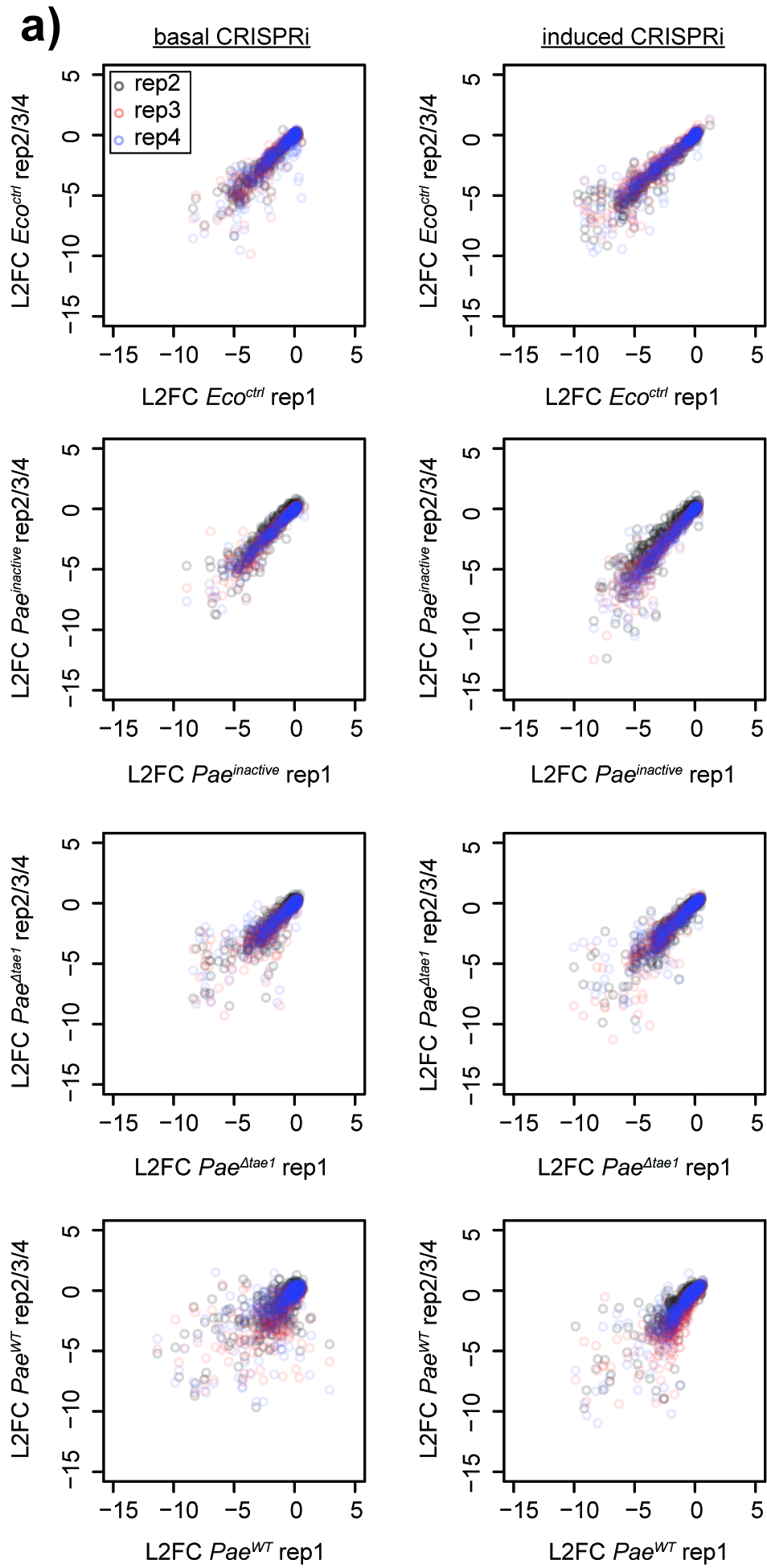

Supplement: S3 Fig — a) CRISPRi library fitness in T6SS screen is reproducible across biological replicates. Replica plots showing the uncorrected L2FC values for each Eco CRISPRi strain after competition against PaeWT, Pae Δtae1, Paeinactive, for four biological replicates. For each plot, replicate 1 is compared to replicate 2 (grey), replicate 3 (red), or replicate 4 (blue). Median Pearson’s r between all replicates = 0.91. (TIF) [file ppat.1011454.s007.tif]

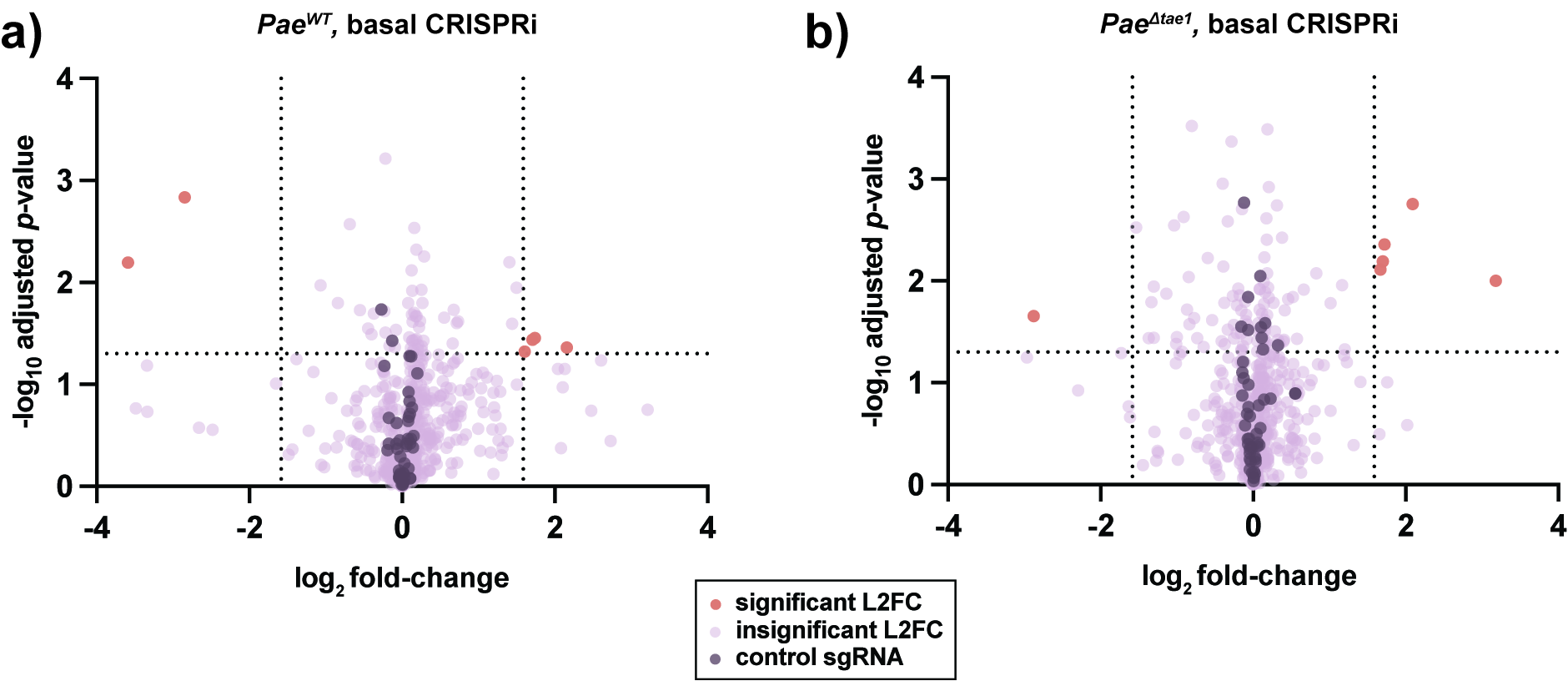

Supplement: S4 Fig — a-b) Basal CRISPRi attenuates Eco fitness phenotypes against PaeWT (a) and PaeΔtae1(b). Volcano plots showing log2-fold change (L2FC) values for each KD strain after interbacterial competition (basal CRISPRi). Data shown: mean from four biological replicates. Statistical test: Wald test. Vertical dotted lines indicate arbitrary cutoffs for L2FC at x = -1.58 and x = 1.58 (absolute FC x = -3 or x = 3). Horizontal dotted line indicates statistical significance cutoff for log10 adjusted p-value (≤ 0.05). Red points represent KDs with L2FC ≥ 1.58 or ≤ -1.58 and log10-adj. p-value ≤0.05. Dark purple points represent non-targeting negative control KDs (n = 50). Lavender points represent KDs that do not meet cutoffs for L2FC or statistical test. (TIF) [file ppat.1011454.s008.tif]

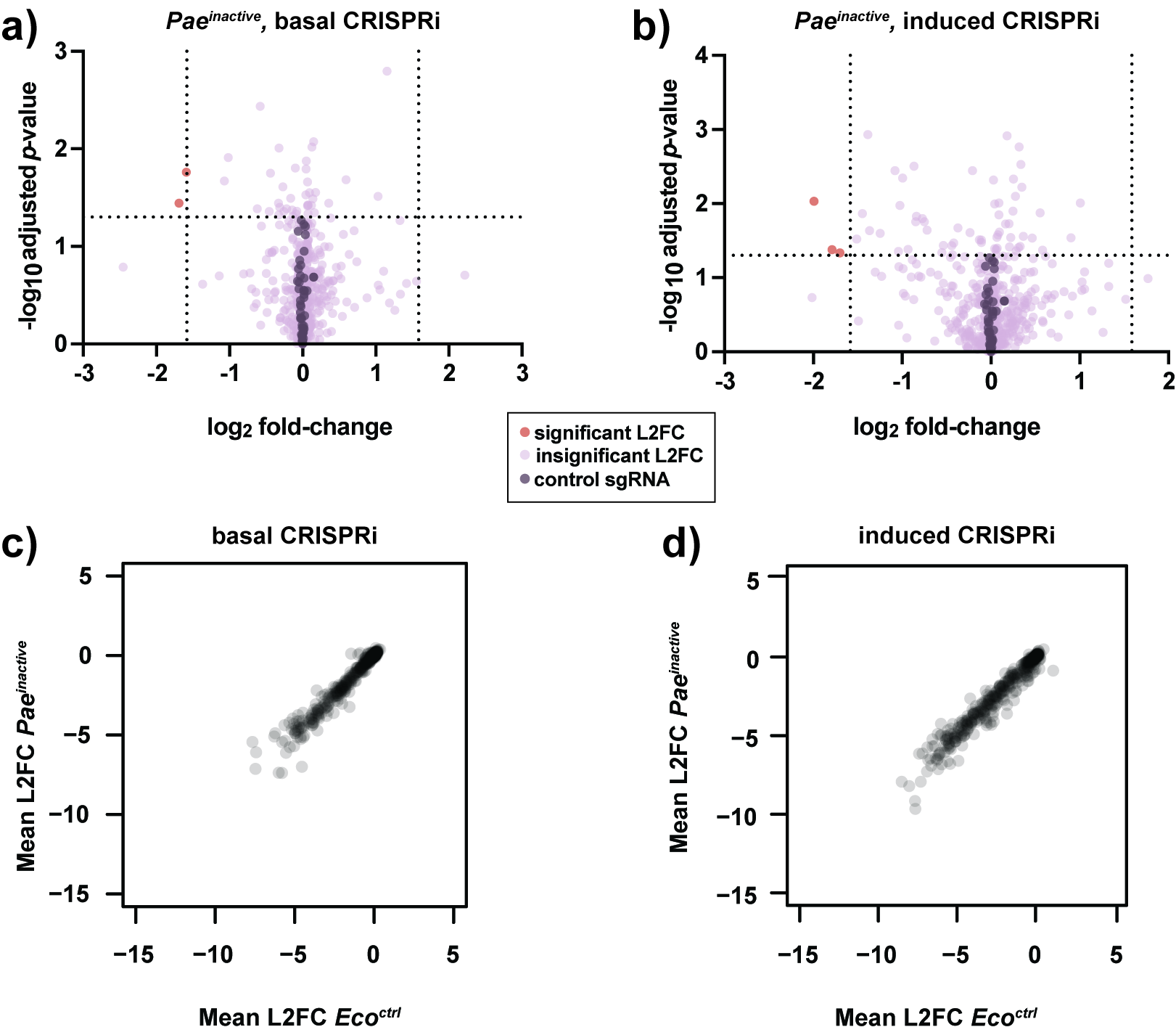

Supplement: S5 Fig — a-b) Competition against Paeinactive reveals few Eco fitness determinants. Volcano plots showing log2-fold change (L2FC) values for each KD strain after interbacterial competition with induced (a) or basal (b) CRISPRi. Data shown: mean from four biological replicates. Statistical test: Wald test. Vertical dotted lines indicate arbitrary cutoffs for L2FC at x = -1.58 and x = 1.58 (absolute FC x = -3 or x = 3). Horizontal dotted line indicates statistical significance cutoff for log10 adjusted p-value (≤ 0.05). Red points represent KDs with L2FC ≥ 1.58 or ≤ -1.58 and log10-adj. p-value ≤0.05. Dark purple points represent non-targeting negative control KDs (n = 50). Lavender points represent KDs that do not meet cutoffs for L2FC or statistical test. c-d) KD strain abundance is highly similar after competition with Paeinactive and after growth without competition (Ecoctrl). Scatter plots comparing mean L2FC for each Eco KD strain after competition with Paeinactive or Ecoctrl treatment, with basal (c) or induced (d) CRISPRi. Median Pearson correlation r = 0.98. (TIF) [file ppat.1011454.s009.tif]

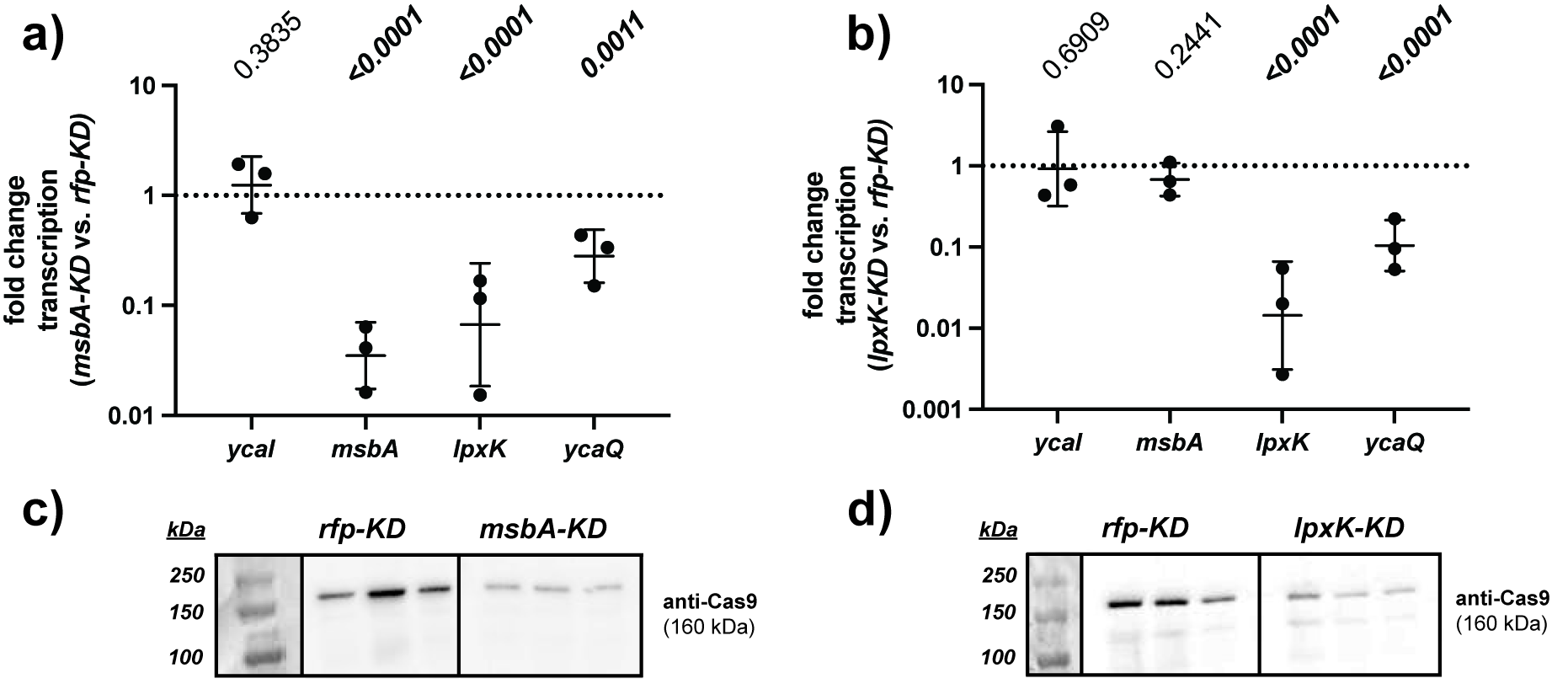

Supplement: S6 Fig — a-b) Transcriptional knockdowns in msbA and lpxK have off-target polar effects on transcription in their operon. qRT-PCR analysis of transcriptional fold-change in ycaI-msbA-lpxK-ycaQ in msbA-KD (a) and in lpxK-KD (b) after growth for 6 hours with induced CRISPRi, normalized to expression in rfp-KD. Data shown are geometric average of 3 biological replicates ± s.d. Statistical test: unpaired two-tailed t-test; p-value ≤0.05 displayed in bold font. c-d) msbA-KD and lpxK-KD express a catalytically dead Cas9 (dCas9) enzyme for CRISPRi-mediated transcriptional knockdown. Western blot analysis of dCas9 protein expression (160 kDa) from rfp-KD, msbA-KD (c), and lpxK-KD (d). Three independent biological replicates shown. (TIF) [file ppat.1011454.s010.tif]

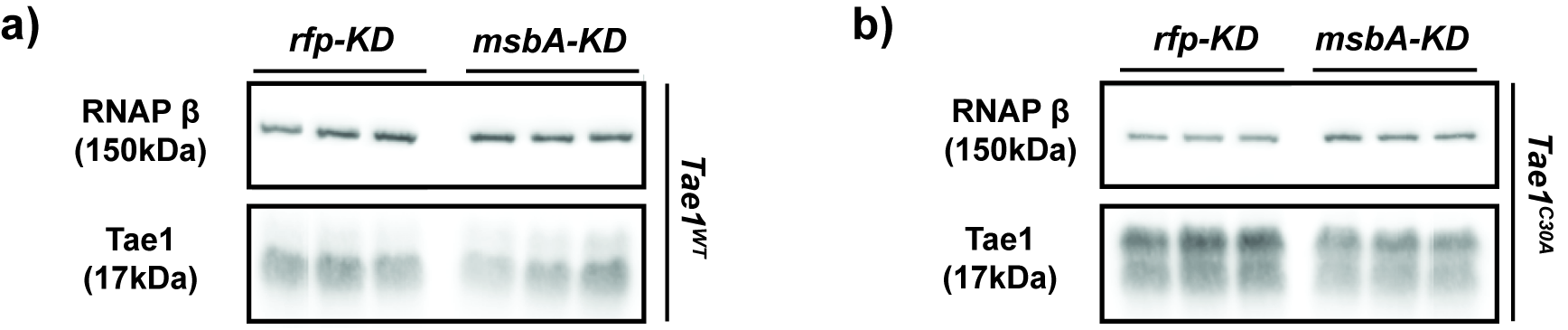

Supplement: S7 Fig — a-b) Bulk Tae1 protein expression is similar between msbA-KD and rfp-KD. Western blot analysis of periplasmic Tae1 protein (17kDa) from (a) pBAD24::pelB-tae1WT (Tae1WT) or (b) pBAD24::pelB-tae1C30A (Tae1C30A) in rfp-KD and msbA-KD (with induced CRISPRi). Protein expression of RNA polymerase (β subunit) (150kDa) is used as an internal loading control. (TIF) [file ppat.1011454.s011.tif]

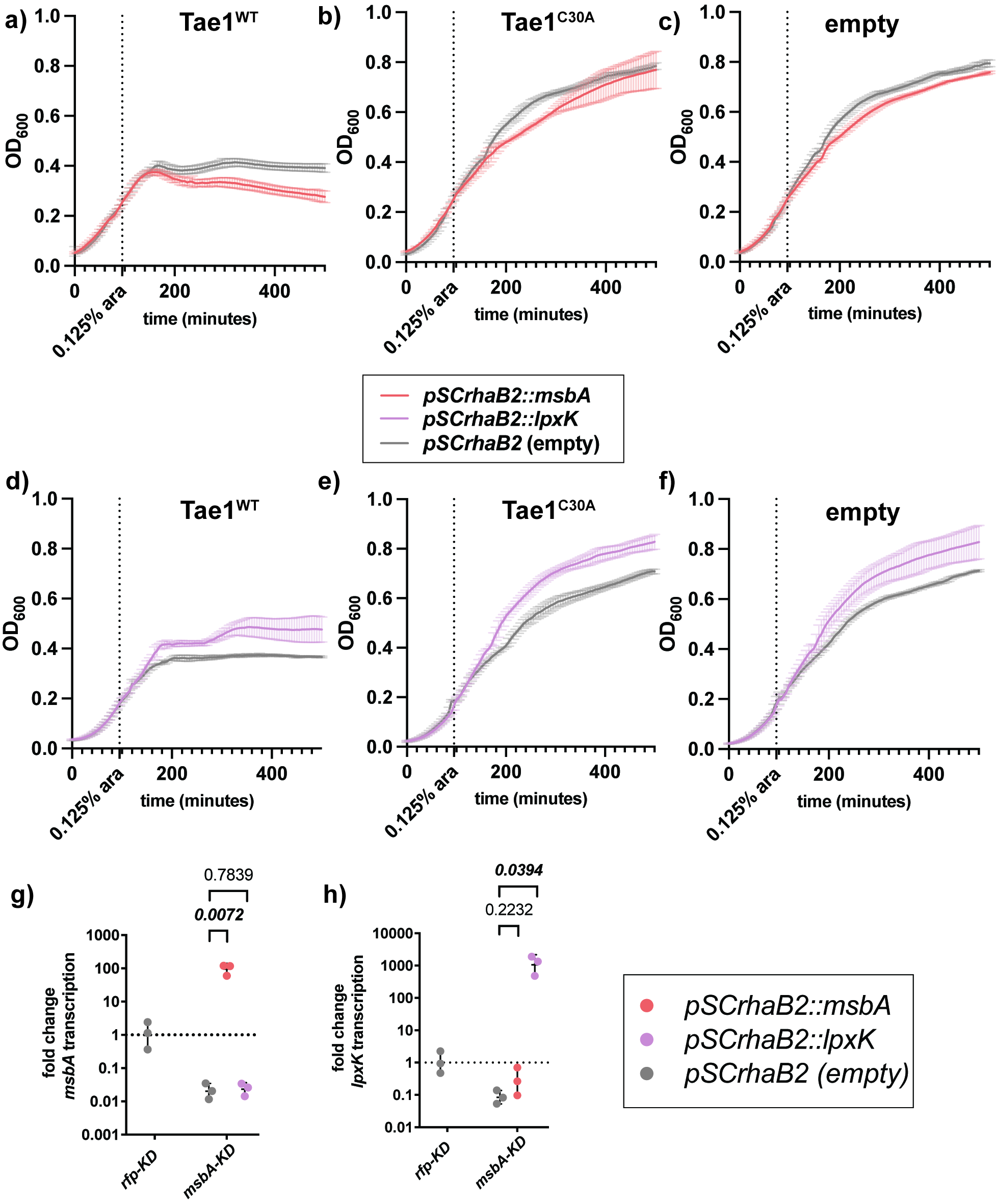

Supplement: S8 Fig — a-c) Plasmid-borne msbA overexpression partially rescues msbA-KD resistance to lysis by Tae1. OD600 growth curves of msbA-KD with induced CRISPRi, overexpressing pSCrhaB2::msbA (red) or pSCrhaB2 (empty) (grey) alongside (a)pBAD24::pelB-tae1WT (Tae1WT), (b) pBAD24::pelB-tae1C30A (Tae1C30A), or (c) pBAD24 (empty). Data shown: average of 3 biological replicates ± s.d. Dotted vertical line indicates pBAD24 induction timepoint (at OD600 = 0.25) (0.125% arabinose w/v). d-f) Plasmid-borne lpxK overexpression enhances msbA-KD resistance to lysis by Tae1. OD600 growth curves of msbA-KD with CRISPRi induced, overexpressing pSCrhaB2::lpxK (purple) or pSCrhaB2 (empty) (grey) alongside (d)pBAD24::pelB-tae1WT (Tae1WT), (e) pBAD24::pelB-tae1C30A (Tae1C30A), or (f) pBAD24 (empty). Data shown: average of 3 biological replicates ± s.d. Dotted vertical line indicates pBAD24 induction timepoint (at OD600 = 0.25) (0.125% arabinose w/v). g-h) pSCrhaB2 vectors selectively rescue transcription of their target gene by overexpression. qRT-PCR analysis of transcriptional fold-change in (g)msbA or (h)lpxK expression with constitutive rhamnose induction of pSCrhaB2::msbA (red), pSCrhaB2::lpxK (purple), or (c)pSCrhaB2 (empty; grey) in msbA-KD with induced CRISPRi. Expression normalized against basal msbA expression in rfp-KD + pSCrhaB2 (empty). Data shown: geometric average of 3 biological replicates ± s.d. Statistical test: unpaired two-tailed t-test; p-value ≤0.05 displayed in bold font. (TIF) [file ppat.1011454.s012.tif]

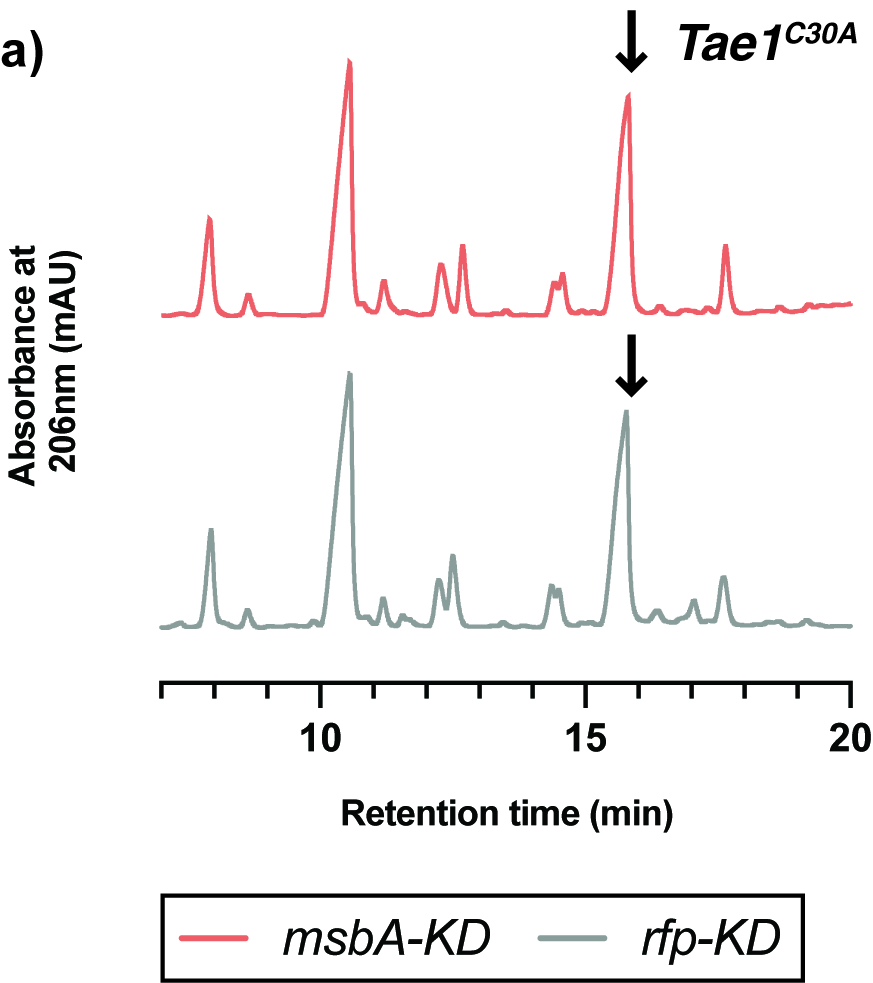

Supplement: S9 Fig — a)Tae1C30A overexpression yields minor digestion of D44 muropeptides. HPLC chromatograms of muropeptides purified from msbA-KD (red) and rfp-KD (grey) expressing pBAD24::pelB-tae1C30A (Tae1C30A). Black arrow indicates D44 peptide partially digested by Tae1C30A. Data shown: representative from 3 biological replicates. (TIF) [file ppat.1011454.s013.tif]

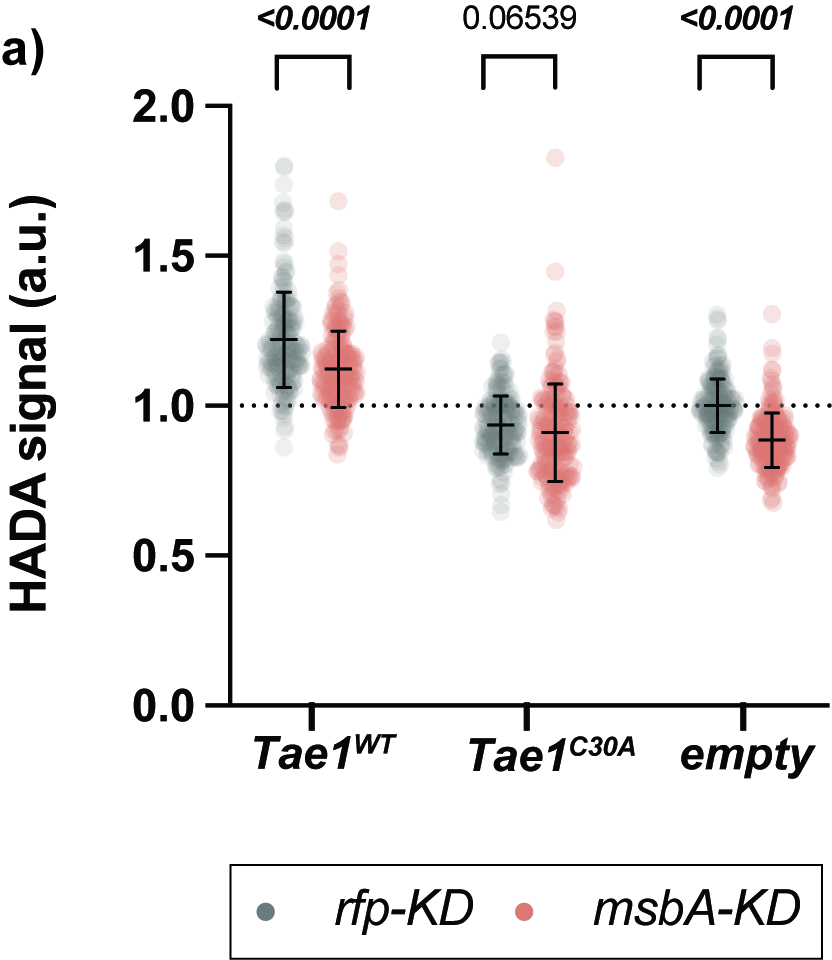

Supplement: S10 Fig — a) PG synthesis activity in msbA-KD is attenuated under all tested conditions. Single-cell fluorescence intensity measurements for rfp-KD (grey) or msbA-KD (red) incorporating the fluorescent d-amino acid HADA into PG after 60 minutes of overexpressing pBAD24::pelB-tae1WT(Tae1WT), pBAD24::pelB-tae1C30A (Tae1C30A), or pBAD24 (empty), with CRISPRi induced. All data normalized to average HADA signal in rfp-KD + empty. Data shown: 600 cells (200 cells x 3 biological replicates), with average ± s.d. Statistical test: unpaired two-tailed t-test; p-value ≤0.05 displayed in bold font. (TIF) [file ppat.1011454.s014.tif]

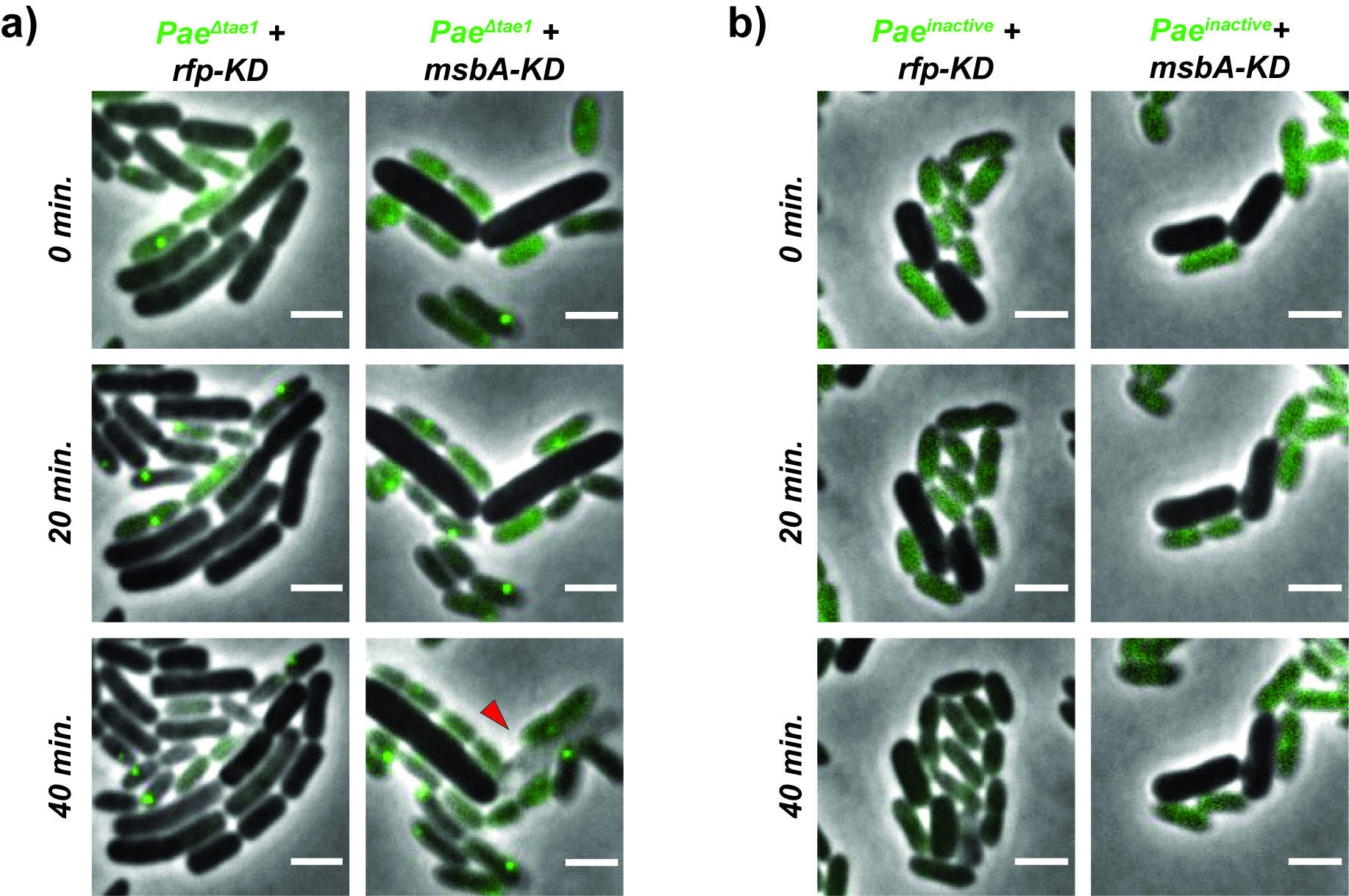

Supplement: S11 Fig — a-b) msbA-KD cells maintain growth defects regardless of Pae competitor. Representative frames from time-course imaging of rfp-KD (left column; grey cells) and msbA-KD (right column; grey cells) co-cultured with Pae Δtae1(a) or Pae inactive (b) (green cells), and with induced CRISPRi. Green foci in PaeWT indicate accumulations of GFP-labelled ClpV, which signal H1-T6SS firing events. Red arrow indicates lysed cell. Data shown are merged phase contrast and fluorescence channels. Scale bar: 2μm. (TIF) [file ppat.1011454.s015.tif]
