# Supplementary material for: Lipopolysaccharide transport regulates bacterial sensitivity to a cell wall-degrading intermicrobial toxin
Source: PLoS Pathog. 2023 Jun 26;19(6):e1011454. doi: 10.1371/journal.ppat.1011454 (PMC10328246; doi:10.1371/journal.ppat.1011454)
Supplement: Attachment — Submitted filename: 133865_1_rebuttal_2651427_rt4bb5.pdf [file ppat.1011454.s016.pdf]

Full Revision

Review COMMONS

Manuscript number: RC-2023-01859
Corresponding author(s): Seemay Chou

1. General Statements

We thank the reviewers for their constructive feedback on our manuscript. They did a very comprehensive and helpful job of laying out some key areas that could be improved. We were heartened by the fact that there was a fair amount of overlap between the two reviewers, and that comments were largely addressable without further experimentation.

Below, we provide a summary of how we have attempted to address the comments and concerns from both reviewers. We also provide the rationale and action items for our responses. Overlapping comments from both reviewers have been consolidated and responded to together.

2. Point-by-point description of the revisions

Comment 1 (Reviewer #1, Minor Comment 1 & Reviewer #2, Significance)

Both reviewers raised concerns about our choice to focus on essential genes in our CRISPRi screen, which could potentially underestimate the role of non-essential factors contributing to Tae1 sensitivity or resistance.

Rationale: We agree with the reviewers that including non-essential genes could provide additional insights into the roles of non-essential factors in Tae1 sensitivity and resistance. We believe our focus on essential genes contributes a unique perspective to the field, as there already exists a body of work that interrogates non-essential genes in this space. Here are some citations that represent this body. We will highlight these better in the manuscript.

Lin, H.-H.; Yu, M.; Sriramoju, M. K.; Hsu, S.-T. D.; Liu, C.-T.; Lai, E.-M. A High-Throughput Interbacterial Competition Screen Identifies ClpAP in Enhancing Recipient Susceptibility to Type VI Secretion System-Mediated Attack by Agrobacterium Tumefaciens. *Front Microbiol* **2020**, *10*, 3077. https://doi.org/10.3389/fmicb.2019.03077.

Hersch, S. J.; Sejuty, R. T.; Manera, K.; Dong, T. G. *High Throughput Identification of Genes Conferring Resistance or Sensitivity to Toxic Effectors Delivered by the Type VI Secretion System*; preprint; Microbiology, 2021. https://doi.org/10.1101/2021.10.06.463450.

Additionally, our screen was experimentally optimized for essential genes using our approach. The knockdown strategy is useful specifically for essential genes because *E.coli* is phenotypically very sensitive to essential gene perturbations (see more here: https://doi.org/10.1128/mBio.02561-21). While it would have been ideal to include non-essential genes too, doing so would require a different additional optimization that we believe would have diluted our bandwidth for this study. We do thank the reviewers for recognizing how much effort went into this!

We do acknowledge this is a limitation and want to make sure the readership is aware of that. Ideally, one could do more rigorous side-by-side comparisons between studies if the approaches, set-up, and assays are the same. Unfortunately, due to differences in experimental set-up, we could not directly compare with the non-essential screens. We hope others will pick up where we left off. Here are some action items we can take to increase the odds of that:

- In the Introduction, we will mention other studies and highlight the need to investigate essential genes side-by-side with non-essential. (Lines 64-7)
- In the Discussion, we will add a sentence that acknowledges the importance of exploring non-essential genes for a more comprehensive understanding of Tae1 sensitivity and resistance. (Lines 484-5)

Comment 2 (Reviewer #1, Minor Comment 5 & Reviewer #2, Major Comment)

Both reviewers mentioned that the dormancy state in *msbA-KD* cells is not well characterized and its relationship with Tae1 resistance is not convincingly shown.

Rationale: We agree that our manuscript does not clearly pin down whether Tae1 resistance is linked to a true dormancy state. There are some intriguing similarities between what we observe and what is classically known as “dormancy” or “persistence”, which have specific definitions. Although we don’t yet have a concrete reason to think it’s NOT those states, we also don’t have sufficient data to point to it clearly being the same at a mechanistic or cellular level. This is merely a hypothesis that our work suggests. We would love to see others follow up on this, as we suspect there are overlaps and potentially additional cellular states that have yet to be clearly defined in this field of bacterial physiology.

Here is how we propose to address this concern:

- We simplified our language to be more descriptive and less loaded in terms of nomenclature around dormancy or persistence. Namely, we are referring to the cells in a more descriptive way with “slowed growth.” This allows us to clearly describe what we observe without attempting to ascribe mechanism or anything beyond that. It doesn’t fundamentally change the overarching interpretation of our study. (Lines 444, 490,497-9)
- In the Discussion, we will add text emphasizing the need for follow-up studies to fully address whether there is indeed a connection between Tae1 resistance and slowed growth. (Lines 491-3)

Comment 3 (Reviewer #2, Major Comment)

The reviewer asks if the degradation of the sugar backbone is also required for lysis or if it is just the crosslinking step that is important.

Rationale: This is an astute point. We acknowledge that the degradation of the sugar backbone may play a role in lysis, and it’s predicted that this may be why the *Pae* H1-T6SS delivers a second PG-degrading toxin (Tge1), a muramidase that targets the sugar backbone. The most parsimonious conclusion from past studies by us and others is that Tae1 is critical for lysis, but not sufficient in the absence of any backbone-targeting enzyme. Indeed, many T6SS-encoding

bacterial species also encode >1 type of PG-degrading enzyme, which may speak precisely to the reviewer's point. However, it should also be noted that there may be endogenous enzymes with activities that can be leveraged alongside these toxins for the same effect.

Action items:

- In the Discussion, we will add a sentence addressing the potential role of sugar backbone degradation in the lysis process and the need for future research on this topic. (Lines 524-6)

Comment 4 (Reviewer #1, Minor Comment 2)

The reviewer asks why *lptC-KD* leads to sensitivity to Tae1, while *msbA-KD* leads to resistance, considering both genes are implicated in LPS export.

Rationale: We appreciate the reviewer's careful attention to the underlying biology. They are absolutely correct in pointing this difference out. Our interpretation is that the different phenotypes may indicate that although the LPS biosynthesis superpathway intersects with PG synthesis, *lptC* and *msbA* may intersect with PG synthesis in distinct ways. We can address this concern through the following:

- We will add a sentence in the Discussion section providing our interpretation of the different phenotypes observed for *lptC-KD* and *msbA-KD*. (Lines 508-13)

Comment 5 (Reviewer #1, Minor Comment 4)

The reviewer notes that the contribution of *msbA* to Tae1 resistance appears minor based on the results in Figure 3d.

Rationale: There are actually two aspects to this concern, which we note below. We found it difficult to fully capture it in the manuscript, but our thoughts are as follows.

(1) Technical viewpoint:

Bacterial competition experiments are inherently noisy. The quantitative read-out is easily impacted by a number of parameters, including cellular density, input ratio between competitor cell types, growth stage, and possibly other environmental factors that are difficult to predict. In general, our view is that we should avoid over-indexing on the *degree* of the phenotype, focusing more on the *direction* of the phenotype (loss of statistically-significant Tae1 sensitivity) and the fact that it is reproducible in our hands. Furthermore, our argument is bolstered by clear validation of the loss of Tae1 sensitivity through orthogonal lysis assays (Fig. 4a-c).

(2) Biological viewpoint

It is challenging to isolate the specific interaction between Tae1 and individual genetic determinants, as we think it's a complex system with multiple factors simultaneously at play. It is crucial to acknowledge that the unique contribution of Tae1 is only a part of the T6SS. There

may be other compensatory actions that influence the outcomes observed, such as upregulation of non-Tae1 toxins, regulation of system activation/firing, timing and location of T6S injections, etc. We think these are exciting possibilities and that more groups should delve into the context-dependent dynamics of the system. Although outside the scope of our manuscript, we would be open to suggestions for how we can further emphasize this point.

Comment 6 (Reviewer #2, Minor Comment)

The reviewer recommends that we discuss whether our findings are specific to Tae1 or if they can be extrapolated to other toxins.

Rationale: We understand the reviewer's interest in understanding the broader implications of our findings. Although our study focuses specifically on Tae1, we believe that our findings may provide insights into the mechanisms of sensitivity and resistance to other toxins that target the cell wall. However, experimentally investigating this would fall outside the scope of our current manuscript.

Additional Minor Revisions

1. Table 1: I would label MsbA and LptC as "LPS transport" and not "LPS synthesis" (Reviewer 1)

   Rationale: We agree that using “LPS transport” to describe the gene functions for lptC and msbA is more specific to their functions.

   - Table 1 was updated to change the “pathway/process” categorizations for lptC and msbA from “LPS synthesis” to “LPS transport”.
   - In line with this comment, we also changed the pathway/process categorization for murJ (Lipid II flippase) to “PG transport”.

2. Figure 3 legend: "...deformed membranes ....are demarcated in (g) and (h)" (Reviewer 1)

   We thank the reviewer for pointing out the missing text in this figure legend.

   - We corrected the error by adding the missing text back in Figure 3.

3. Line 339-341: Supp. Fig. 9 should be Supp. Fig. 8 (Reviewer 1)

   - *Referenced Supp. Fig. was corrected.*

4. Second, (L422-425) the authors conclude that their data demonstrate a "reactive crosstalk between LPS and PG synthesis". I disagree. There is no information in the paper that this is the case. The authors can only suggest that cross talk may occur. (Reviewer 2)

- *We agree. Line 421-2: replaced "demonstrate" with "suggest" to soften the argument.*
