# Supplementary material for: Lipopolysaccharide transport regulates bacterial sensitivity to a cell wall-degrading intermicrobial toxin
Source: PLoS Pathog. 2023 Jun 26;19(6):e1011454. doi: 10.1371/journal.ppat.1011454 (PMC10328246; doi:10.1371/journal.ppat.1011454)
Supplement: Attachment — Submitted filename: 23_Trotta_CoverLetter_PLOSPathogens_revision.docx.pdf [file ppat.1011454.s017.pdf]

UCSF
University of California
San Francisco

Seemay Chou, PhD
Adjunct Assistant Professor
Department of Biochemistry and Biophysics
seemaychou@gmail.com

May 18, 2023

To: Managing Editor, *PLOS Pathogens*

We are submitting a revised version of our manuscript, which has been retitled to "Lipopolysaccharide transport regulates bacterial sensitivity to a cell wall-degrading intermicrobial toxin" for consideration at *PLOS Pathogens.* Below we have summarized the key changes that our revised manuscript includes.

1. Title change

In response to feedback for more precision in our title, we have revised it to focus more on LPS transport rather than LPS in general. The current title is now: "Lipopolysaccharide transport regulates bacterial sensitivity to a cell wall-degrading intermicrobial toxin"

2. Abstract revision

To clarify some of our assertions in our abstract, we made changes to two sentences. First, for the sentence beginning "Disruption of.." (line 22), we added in language to specify the stage of LPS synthesis under consideration. Second, for the final sentence (lines 27-28) we modified the language and streamlined its syntax for simplicity and clarity.

3. Funding source revision

We caught an error regarding Basler's funding, and have revised this section for accuracy.

Thank you in advance for your efforts and consideration. Please let me know if you have any additional questions or concerns. We appreciate your feedback and help on this matter.

Sincerely,

Seemay Chou, PhD
