# Supplementary material for: Lipopolysaccharide transport regulates bacterial sensitivity to a cell wall-degrading intermicrobial toxin
Source: PLoS Pathog. 2023 Jun 26;19(6):e1011454. doi: 10.1371/journal.ppat.1011454 (PMC10328246; doi:10.1371/journal.ppat.1011454)
Supplement: Peer review history

# Author comment: Author response to previous submission

**DOI:** 10.1371/journal.ppat.1011454.r001

2 May 2023

Attachment Submitted filename: 133865_1_rebuttal_2651427_rt4bb5.pdf Click here for additional data file.

# Editor report: Decision Letter 0

**DOI:** 10.1371/journal.ppat.1011454.r002

**Authors:** Nina R. Salama

6 May 2023

Dear Dr Chou,

Thank you very much for submitting your manuscript "Lipopolysaccharide integrity primes bacterial sensitivity to a cell wall-degrading intermicrobial toxin" with revisions directed by reviews from Reviews Commons for consideration at PLOS Pathogens.  While a few concerns remains, we are likely to accept this manuscript for publication, providing that you modify the manuscript according to the concerns raised during editorial review.

While the revisions undertaken in response to the Review Commons review seem reasonable, the conceptual issues raised by the reviewers require some modification to the title and abstract to be consistent with the data reported. Please address the following:

1. Title: LPS integrity has not been assessed in this manuscript (except for indirectly through EM). Furthermore, as pointed out in the manuscript, some LPS synthesis/transport mutants give sensitivity while others give resistance. Figures 5 and 6 suggest that for the MsbAKD strain, the phenotype results from a homeostatic slow growth response as measured by lower protein synthesis and cell wall incorporation. Whether other KD strains targeting the LPS synthesis pathway show a similar response (or whether the sensitive mutants do not slow growth) has not been investigated. To keep the current title more experiments will be needed. Alternatively the title should be changed to focus more on MsbA or perhaps "Perturbation of LPS synthesis modulates bacterial sensitivity..."

2. Abstract: Similarly the last line of the abstract seems too grandiose (and I'm not sure even what it means). The data could suggest that MsbA activity triggers a key cell cycle/growth checkpoint between the LPS synthesis pathway and cell growth, but more experiments would be needed to flesh out this regulatory pathway. Similarly, the data suggest that Tae1 can only target rapidly growing cells, which perhaps is not that surprising. Please modify the last sentence of the abstract to be more clear and to align better with the data presented in the paper.

Please prepare and submit your revised manuscript within 30 days. If you anticipate any delay, please let us know the expected resubmission date by replying to this email.

When you are ready to resubmit, please upload the following:

[1] A letter containing a detailed list of your responses to all review comments, and a description of the changes you have made in the manuscript.

Please note while forming your response, if your article is accepted, you may have the opportunity to make the peer review history publicly available. The record will include editor decision letters (with reviews) and your responses to reviewer comments. If eligible, we will contact you to opt in or out

[2] Two versions of the revised manuscript: one with either highlights or tracked changes denoting where the text has been changed; the other a clean version (uploaded as the manuscript file).

Important additional instructions are given below your reviewer comments.

Thank you again for your submission to our journal. We hope that our editorial process has been constructive so far, and we welcome your feedback at any time. Please don't hesitate to contact us if you have any questions or comments.

Sincerely,

Nina R. Salama

Section Editor

PLOS Pathogens

Nina Salama

Section Editor

PLOS Pathogens

Kasturi Haldar

Editor-in-Chief

PLOS Pathogens

​orcid.org/0000-0001-5065-158X

Michael Malim

Editor-in-Chief

PLOS Pathogens

orcid.org/0000-0002-7699-2064

***********************

Thank you for the opportunity to consider your manuscript. While the revisions undertaken in response to the Review Commons review seem reasonable, the conceptual issues raised by the reviewers require some modification to the title and abstract to be consistent with the data reported. Please address the following:

1. Title: LPS integrity has not been assessed in this manuscript (except for indirectly through EM). Furthermore, as pointed out in the manuscript, some LPS synthesis/transport mutants give sensitivity while others give resistance. Figures 5 and 6 suggest that for the MsbAKD strain, the phenotype results from a homeostatic slow growth response as measured by lower protein synthesis and cell wall incorporation. Whether other KD strains targeting the LPS synthesis pathway show a similar response (or whether the sensitive mutants do not slow growth) has not been investigated. To keep the current title more experiments will be needed. Alternatively the title should be changed to focus more on MsbA or perhaps "Perturbation of LPS synthesis modulates bacterial sensitivity..."

2. Abstract: Similarly the last line of the abstract seems too grandiose (and I'm not sure what it means). The data could suggest that MsbA activity triggers a key cell cycle/growth checkpoint for the LPS synthesis pathway and cell growth, but more experiments would be needed to flesh out this regulatory pathway. Similarly, the data suggest that Tae1 can only target rapidly growing cells, which perhaps is not that surprising. Please modify the last sentence of the abstract to be more clear and to align better with the data presented in the paper.

Reviewer Comments (if any, and for reference):

Figure Files:

While revising your submission, please upload your figure files to the Preflight Analysis and Conversion Engine (PACE) digital diagnostic tool, https://pacev2.apexcovantage.com. PACE helps ensure that figures meet PLOS requirements. To use PACE, you must first register as a user. Then, login and navigate to the UPLOAD tab, where you will find detailed instructions on how to use the tool. If you encounter any issues or have any questions when using PACE, please email us at figures@plos.org.

Data Requirements:

Please note that, as a condition of publication, PLOS' data policy requires that you make available all data used to draw the conclusions outlined in your manuscript. Data must be deposited in an appropriate repository, included within the body of the manuscript, or uploaded as supporting information. This includes all numerical values that were used to generate graphs, histograms etc.. For an example see here: http://www.plosbiology.org/article/info%3Adoi%2F10.1371%2Fjournal.pbio.1001908#s5.

Reproducibility:

To enhance the reproducibility of your results, we recommend that you deposit your laboratory protocols in protocols.io, where a protocol can be assigned its own identifier (DOI) such that it can be cited independently in the future. Additionally, PLOS ONE offers an option to publish peer-reviewed clinical study protocols. Read more information on sharing protocols at https://plos.org/protocols?utm_medium=editorial-email&utm_source=authorletters&utm_campaign=protocols

References:

Please review your reference list to ensure that it is complete and correct. If you have cited papers that have been retracted, please include the rationale for doing so in the manuscript text, or remove these references and replace them with relevant current references. Any changes to the reference list should be mentioned in the rebuttal letter that accompanies your revised manuscript. If you need to cite a retracted article, indicate the article’s retracted status in the References list and also include a citation and full reference for the retraction notice.

# Author comment: Author response to Decision Letter 0

**DOI:** 10.1371/journal.ppat.1011454.r003

25 May 2023

Attachment Submitted filename: 23_Trotta_CoverLetter_PLOSPathogens_revision.docx.pdf Click here for additional data file.

# Editor report: Decision Letter 1

**DOI:** 10.1371/journal.ppat.1011454.r004

**Authors:** Nina R. Salama

1 Jun 2023

Dear Dr Chou,

We are pleased to inform you that your manuscript 'Lipopolysaccharide transport regulates bacterial sensitivity to a cell wall-degrading intermicrobial toxin' has been provisionally accepted for publication in PLOS Pathogens.

Before your manuscript can be formally accepted you will need to complete some formatting changes, which you will receive in a follow up email. A member of our team will be in touch with a set of requests.

Please note that your manuscript will not be scheduled for publication until you have made the required changes, so a swift response is appreciated.

IMPORTANT: The editorial review process is now complete. PLOS will only permit corrections to spelling, formatting or significant scientific errors from this point onwards. Requests for major changes, or any which affect the scientific understanding of your work, will cause delays to the publication date of your manuscript.

Should you, your institution's press office or the journal office choose to press release your paper, you will automatically be opted out of early publication. We ask that you notify us now if you or your institution is planning to press release the article. All press must be co-ordinated with PLOS.

Thank you again for supporting Open Access publishing; we are looking forward to publishing your work in PLOS Pathogens.

Best regards,

Nina R. Salama

Section Editor

PLOS Pathogens

Nina Salama

Section Editor

PLOS Pathogens

Kasturi Haldar

Editor-in-Chief

PLOS Pathogens

​orcid.org/0000-0001-5065-158X

Michael Malim

Editor-in-Chief

PLOS Pathogens

orcid.org/0000-0002-7699-2064

***********************************************************

Reviewer Comments (if any, and for reference):

# Editor report: Acceptance letter

**DOI:** 10.1371/journal.ppat.1011454.r005

**Authors:** Nina R. Salama

22 Jun 2023

Dear Dr Chou,

We are delighted to inform you that your manuscript, "Lipopolysaccharide transport regulates bacterial sensitivity to a cell wall-degrading intermicrobial toxin," has been formally accepted for publication in PLOS Pathogens.

We have now passed your article onto the PLOS Production Department who will complete the rest of the pre-publication process. All authors will receive a confirmation email upon publication.

The corresponding author will soon be receiving a typeset proof for review, to ensure errors have not been introduced during production. Please review the PDF proof of your manuscript carefully, as this is the last chance to correct any scientific or type-setting errors. Please note that major changes, or those which affect the scientific understanding of the work, will likely cause delays to the publication date of your manuscript. Note: Proofs for Front Matter articles (Pearls, Reviews, Opinions, etc...) are generated on a different schedule and may not be made available as quickly.

Soon after your final files are uploaded, the early version of your manuscript, if you opted to have an early version of your article, will be published online. The date of the early version will be your article's publication date. The final article will be published to the same URL, and all versions of the paper will be accessible to readers.

Thank you again for supporting open-access publishing; we are looking forward to publishing your work in PLOS Pathogens. 

Best regards,

Kasturi Haldar

Editor-in-Chief

PLOS Pathogens

​orcid.org/0000-0001-5065-158X

Michael Malim

Editor-in-Chief

PLOS Pathogens

orcid.org/0000-0002-7699-2064
